# Comparison of Dynamic Light Scattering and Rheometrical Methods to Determine the Gel Point of a Radically Polymerized Hydrogel under Mechanical Shear

**DOI:** 10.3390/mi11050462

**Published:** 2020-04-28

**Authors:** Katinka Kohl

**Affiliations:** Chair of Magnetofluiddynamics, Measuring and Automation Technology, Technische Universität Dresden, 01069 Dresden, Germany; katinka.kohl@tu-dresden.de

**Keywords:** rheometry, dynamic light scattering (DLS), hydrogel, gel point, mechanical shear

## Abstract

The phase transition of nanocomposite hydrogels made of N-isopropylacrylamide (NIPAm) and clay (Laponite^®^ XLS) was investigated under mechanical shear influencing the gelation. The hydrogels were synthesized by free radical polymerization. For the processing of cross-linked gels, the phase transition (liquid–solid) and its dependence on mechanical stress are of paramount importance. On the one hand, the determination of the gel point (*tg*) is possible with rheometry and, on the other hand, with dynamic light scattering (DLS). With rotational rheometry, by identifying the abrupt increase of viscosity, the gel point is evaluated. The DSL is an alternative method to rheometry, to investigate hydrogels under the action of the shear flow, to make results comparable to the rheometric investigations, with and without shear. Experimental parameters were chosen based on preparatory work to obtain comparable results regarding the determination of the gel point of a radically polymerized NIPAm hydrogel.

## 1. Introduction

There has been intense interest and progress in engineering soft, shape-transforming materials, such as hydrogels. Polymer hydrogels responsive to stimuli have attracted increasing attention as promising soft materials because they can have significant volume changes in response to external stimuli. The main focus of research is on polymers that change their physical properties drastically and discontinuously with temperature. Mainly, polymers that show thermosensitivity in aqueous solutions [1,2,3]. The possible fields of application for thermoresponsive polymers are tissue engineering [4], chromatography [5], drug release [6], bioseparation [7], sensor and actuator materials for specialized applications [8].

In the technical application of this new class of materials, hydrogels, with their viscoelastic behavior, the main focus is on improving the mechanical properties. Haraguchi et al. [9,10,11] reported on soft hydrogels based on N-isopropylacrylamide (NIPAm) with exceptional tensile properties and mechanical toughness. The hydrogels based on NIPAm are used for various applications and are the most studied systems [1]. The NIPAm hydrogels with covalently cross-linked network structures are radically polymerized. The polymerization is initiated by the redox system potassium persulfate (PPS) and N,N,N′,N′-tetramethylethylendiamine (TEMED) in the presence of clay the Laponite^®^ XLS (synthetic layered silicate and platelets) [12,13,14]. The combination of clay (inorganic) with polymer (organic) is called nanocomposite hydrogel (NC hydrogel). The NC hydrogels used in this work are chemical gels as well as based on the organic NIPAm polymer and inorganic clay nanoparticles [15].

The clay platelets in an aqueous solution act as a polyfunctional crosslinker of movable polymer chains. These NC hydrogels are attractive for technical applications because of their improved mechanical stability, compared to hydrogels without synthetic layered silicate. Shibayama et al. concluded that “the high mechanical properties of NC gels are ascribed to “plane cross-linking” with long PNIPA chains between platelets compared with those of conventional chemical gels having “point cross-linking”. The generally accepted gel formation mechanism in the NIPAm–clay hybrid [12,13,14,15,16] involves the formation of the “clay-brush particles”, i.e., clay aggregates with polymer chains adsorbed on the surface of clay platelets, in the beginning of polymerization. Clay aggregates lead to a reduction of optical transmittance (turbidity). Linking of these clay brushes takes place to form large assemblies [12,17,18] and hydrogel clusters followed by gelation of the system. When the gel point (*tg*) is reached, turbidity appears in the clear polymer solution. Continuation of the polymerization/gelation and formation of long grafted chains result in a gradual homogenization and an increase in the transparency of a system. The model of the gelation mechanism is based on measurements of viscosity in the early pregel stage [19], changes in transparency during polymerization, and dynamic light scattering (DLS) [20]. In particular, the effect of mechanical shear on the formation and properties of a polymer/clay gel is still not understood.

As it is known, the gel point can be evaluated using rotational and oscillating rheometry [21]. The intersection of storage modulus G′ and loss modulus G″ is used in the oscillating test to define the gel point at which a higher storage modulus compared to the loss modulus is observed (G′ > G″) [22,23,24,25,26,27]. Using rotational rheometry, gel point is evaluated by identifying the abruptly increase of the viscosity. The results of the rotation measurements provide a possibility to find a critical mechanical shear (shear stress), which significantly influences the gel formation. Here, too, it could be shown that mechanical stress during the phase transition has influences on the gelling time and, thus, on the mechanical stability of the final hydrogel. For the NIPAm hydrogel with clay (Laponite^®^ XLS) used as cross-linker, rotation and oscillation measurements under a mechanical load have already been successfully carried out in the past [27]. This work aims to compare rotational rheometry with dynamic light scattering for measuring the gelation time in NC hydrogels to observe the mechanical shear influence on the gel point. As the preparatory work [27] having shown, mechanical stress influences on the gelling time. The gelling time without shear can only be determined with dynamic light scattering and tilting tests. Therefore, the rheometric methods were compared with the DLS, which is an alternative to examine the gel point without mechanical shear. The first studies on polymer networks using DLS were carried out on gelatine gels several decades ago [28]. Gelling using DLS is indicated by changing the form of the time–intensity correlation function (TCF or ICF). The TCF reveals a power-law behavior at the gel point [16,29,30,31]. The unique feature of our current study was the measurement with the DLS under shear flow. A self-made setup, allows determining the gel point in the resting and dynamic state using a combination of two technically independent measuring techniques for the first time. With the help of extensive sensitivity analysis in preparatory work, concrete measurement parameters were set, which are required to obtain reproducible results. These rheometric results serve as the basis for the comparison with the DLS.

## 2. Materials and Methods

### 2.1. Materials

N-isopropylacrylamide (NIPAm) monomer (stabilized with 4-Methoxyphenol (MEHQ)) was purchased from TCI Chemicals (Eschborn, Germany) with a purity of >98%. Potassium persulfate (PPS, Sigma Aldrich, St. Louis, MO, USA), N,N,N′N′-tetramethylethylenediamine 99.5% (TEMED, Sigma Aldrich) and Laponite^®^ XLS was purchased from BYK Additives and Instruments (Wesel, Germany). The gels were prepared and equilibrated in Milli-Q deionized water (DIW–H_2_O). The prepared solutions were deoxygenated by argon before polymerization.

### 2.2. Hydrogel Polymerization

The gelation of the hydrogel based on NIPAm and clay (Laponite^®^ XLS) was based on a free radical polymerization with potassium persulfate (PPS) and TEMED [9,29]. The starting solution consisted of 3 g Laponite^®^ XLS (approximately 40 wt % in dry gel) were dispersed in 40 mL DIW–H_2_O under constant stirring for 15 h. Then 5 g NIPAm were added, and the mixture was stirred for another two hours. TEMED was dissolved in DIW–H_2_O under constant stirring for two hours to achieve a concentration of 0.6 mol/L. Furthermore, the initiator, PPS, was dissolved in DIW–H_2_O to achieve a concentration of 0.036 mol/L [27]. A molar ratio with a sample volume of 1 mL (NIPAm): (TEMED): (KPS) = 8.8 × 10^−4^: 6 × 10^−5^: 3.7 × 10^−6^ was used.

### 2.3. Methods

Rheometric measurements were performed with an Anton-Paar Physica MCR 301 rheometer (Anton Paar, Nuremberg, Germany) equipped with cone-plate geometry (diameter: 50 mm, opening angle: 0.995°). To perform the measurements, the three components (NIPAm-, PPS- and TEMED-solutions) were mixed: 0.8 mL of NC hydrogel starting solution, 0.1 mL of the solved TEMED and 0.1 mL of the solved PPS. Sample volume of 1 mL (NIPAm): (TEMED): (KPS) = 8.8 × 10^−4^: 6 × 10^−5^: 3.7 × 10^−6^. Subsequently, the mixture was immediately filled into the rheometer gap with a syringe, and we started the measurement. The shear stress was set between 0.5 and 4.5 Pa for the rotational rheometry. The starting point of the given times in the results presented refers to the moment of the mixing. For these experiments, the rheometer was used in the controlled stress mode, avoiding certain fracturing of the gel samples [27]. We performed all measurements at a constant temperature of 293 K ensured by the Peltier unit.

**Figure 1:**
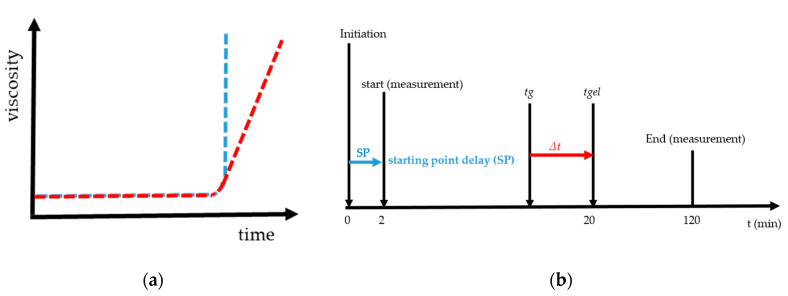
These curves show in (**a**) the fast increase in viscosity over time with a blue curve and a steep increase and red curve a flat increase in viscosity and (**b**) the graphical explanation and relations between delta *t*, gel point (*tg*), gelation time (*tgel*) and starting point delay (SP).

The gel point (*tg*) is the fast increase in viscosity over time and is given graphically in Figure 1a. The blue curve is an example of the steep rise and the red curve with a flat increase of viscosity. Figure 1b gives the graphical overview of starting point delay (SP), gel point (*tg*) and gelation time (*tgel*). SP is the time from mixing, e.g., starting the radical polymerization by initiation with TEMED and PPS until starting the measurement with the rheometer. In the case of a determining gel point with rotational rheometry, it is necessary to define the rate of increase in viscosity. It was established by preliminary research [27] that for low shear stresses, a steep and sudden increase of the viscosity was measured, Figure 1a blue curve. For higher values a slow and flat increase of the viscosity was present, Figure 1a red curve. We found that the definition of the gel point with 1 mPa·s/s at 293 K led to reproducible, unambiguous and comparable results. In addition to *tg* the gelation time (*tgel*) and delta *t* are plotted in Figure 1b. The gel time corresponds to the time from initiation until a macroscopic gel is formed. For the calculation of delta *t*, 20 min was assumed for tgel. Delta *t* is the difference of tgel and *tg*. In [27] the gel point found for very low shear stresses *tg* = 19.6 min. Further details regarding the method and the examination of the gelation using this principle can be found in [27].

**Figure 2:**
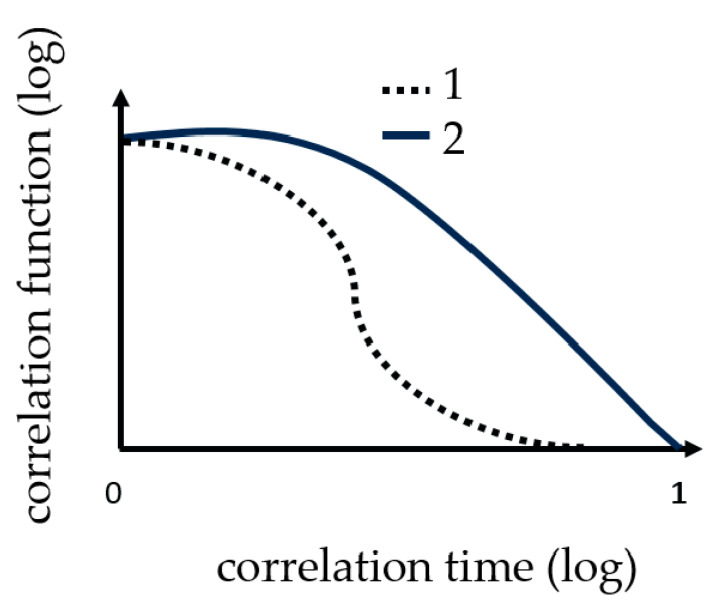
These curves show the change in correlation function (TCF) during the measuring of gelation. 1 the correlation function at the beginning and 2 the correlation function change at the gel point.

Dynamic light scattering measurements were performed with the ZetasizerNano from Malvern, with a HE-NE Laser 633 nm, maximum 4 mW and the new back scattering method (NIBS) [32]. The sample was filled in disposable polystyrene cuvettes and capillary cells for testing under shear flow. The folded capillary cell combines the advantages of the measurement accuracy of a capillary cell with the user-friendliness of a disposable cell. The gel point was determined with DLS, because before the gelation threshold was reached, a change in the DLS autocorrelation functions appeared. Further details regarding the method can be found in [16,20,29,30,31]. Martin et al. demonstrated that the scattering intensity–time correlation function $g_{2}\left(t\right)$ changed from a stretched exponential function to a power-law function, i.e., $g_{2}\left(t\right)-1$ at the gel point [33,34]. The change in the correlation function is shown graphically in Figure 2. 

**Figure 3:**
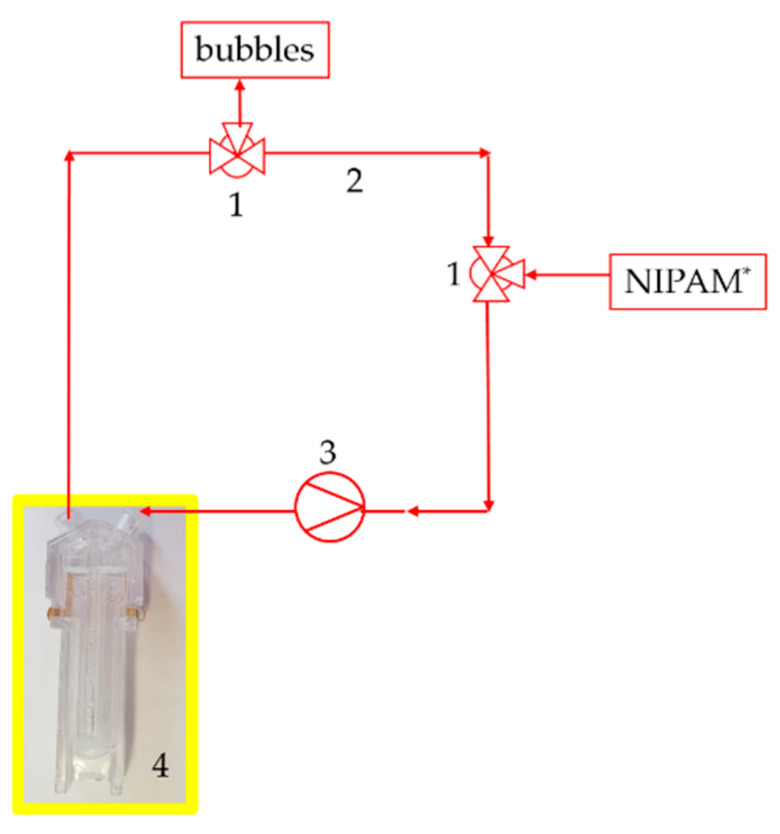
Setup of the dynamic light scattering (DLS) measurement under flow. Two three-way valves (**1**) as input for NIPAm and output for bubbles with the hose (**2**) system, the pump (**3**) and the special cuvette (**4**).

A sample volume of 1 mL was used for the measurements in the resting state. The sample was composed of 800 µL of a 1 mol/L NIPAm solution with dispersed clay (Laponite^®^ XLS) with approximately 40 wt % in dry gel, 100 µL catalyst (TEMED *c* = 0.6 mol/L) and 100 µL initiator (PPS *c* = 0.03 mol/L). A molar ratio with a sample volume of 1 mL (NIPAm): (TEMED): (KPS) = 8.8 × 10^−4^: 6 × 10^−5^: 3.7 × 10^−6^ was used. The measurements under flow were then performed at certain wall shear rates $\dot{\gamma }$ providing shear stress comparable with the rheometric setup. The wall shear rate was set up experimentally with a peristaltic pump via the volume flow $\dot{V}$. A hose peristaltic pump from Medorex was used with a PVA 4.0 × 1.5 hose. TBE/106-1-6 with 106 mm pump head diameter, one channel, 6 mL/min up to 2000 mL/min flow rate and 6 rollers. The measurement setup is given as a schematic sketch in Figure 3. 

To do this, at least 3 calibration measurements were carried out. A sample volume of 60 mL was used for the measurement. The sample consists of 48 mL of a 1 mol NIPAm solution with a mass fraction of approximately 40 wt % of Laponite^®^ XLS in the dry nanocomposite, 6 mL catalyst (TEMED *c* = 0.6 mol/L) and 6 mL initiator (PPS *c* = 0.036 mol/L). The DLS measurements were as well carried out at 293 K. The sample was mixed in a beaker and transferred with a syringe to the flow/hose system. At the same time, the measurement was started at the DLS, because there was a 2-min delay in the start time. The overall measurement time was 30 min, with 5 s per point at 12 points per minute. The digital correlator has generated a correlation function per minute, i.e., the correlation function (1) in Figure 2 would correspond to a measuring time of one minute from 12 measuring points at an interval of 5 s.

It is not possible to control the shear stress in the hose system of the DLS setup. Thus, a wall shear rate ${\dot{\gamma }}_{c}$ (1/s), which corresponds to the shear stress of 0.5 Pa used in rheometry, should be determined and set up in the corresponding DLS experiments. This can be done setting up the volumetric flow $\dot{\gamma }=45$ (mL/min) (Equation (1)) [35]. With a simplified assumption: horizontal tube, stationary and laminar flow, and ideal viscous and incompressible liquid, according to the relationship between Hagen and Poiseuille [35]. Calculated shear rate ${\dot{\gamma }}_{c}$ = 105 (1/s) for a shear stress of 0.5 Pa and the comparable shear rate ${\dot{\gamma }}_{m}$ = 119 (1/s) during real measurement. This shows the difference in shear rates with volume flow specifications. The hose of the DLS setup has a circular cross-sectional area with a radius of *R* = 2 mm. The resulting wall shear rate in the flow experiment ${\dot{\gamma }}_{m}$ is determined experimentally. The volume flow changes due to the speed of the pump and the contact pressure. After we adjusted both on the pump, the actual volume flow is determined using the flow rate using a balance.

(1) $${\dot{\gamma }}_{c}=\frac{4\cdot \dot{V}}{\pi \cdot R^{3}}\;\mathrm{and}\;\dot{V}=\frac{\dot{\gamma }\cdot \pi \cdot R^{3}}{4}$$

A peristaltic pump was used for this, in which the volume flow $\dot{V}$ is to be set manually via the speed control, which leads to some differences compared to the calculated values.

## 3. Results

### 3.1. Determination of the Gel Point Using Rotational Rheometry

**Figure 4:**
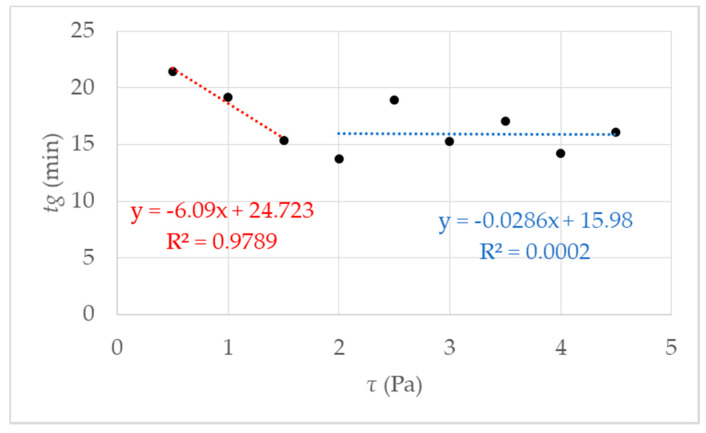
Gel point *tg* measured using rotational rheometry for nine different shear stresses *τ*. The data points represent several independent measurements, meaning different starting solutions and measurement days. The red fit using a linear behavior is for the data points 0.5–1.5 Pa; in blue, using a linear fit between data points 2–4.5 Pa can be regarded as a guide for the eye.

It is an approved method to determine gelling by the fast increase in viscosity over time [27,36]. The rotational rheometry is characterized by a constant rotational speed of the sample geometry during an examination. By determining the gel point, it is necessary to define the rate of increase in viscosity. It was determined by preliminary research [27] that the definition of the gel point at 1 mPa·s/s at 293 K leads to reproducible results. Accordingly, gelling behavior depends on the respective shear stress. Results of measurements using different shear stress *τ* are presented in Figure 4. The respective shear stress *τ* strongly influences the gelation of the investigated hydrogel system. At first glance, it seems paradoxical that the gelation time decreases with increasing shear. As we know from our previous work [27] that the gelation increase with increasing shear. We prepared a measurement series with 9 data points for *τ* = 0.5 up to *τ* = 4.5 Pa. The results are shown in Figure 4.

The first fit in red between data points 0.5 and 2 Pa shows a decrease of *tg*, which leads to the assumption of a critical shear stress, lower than expected 4.75 Pa, as mentioned in [27]. Further investigations were performed and figured out to prove the hypothesis. We made a visual check of the sample after the measurement by opening the measuring set up. With low shear stress, a homogeneous hydrogel film could be observed on the measurement geometry. The proportion of liquid increased with increasing shear stress *τ* > 1.5 Pa. 

**Figure 5:**
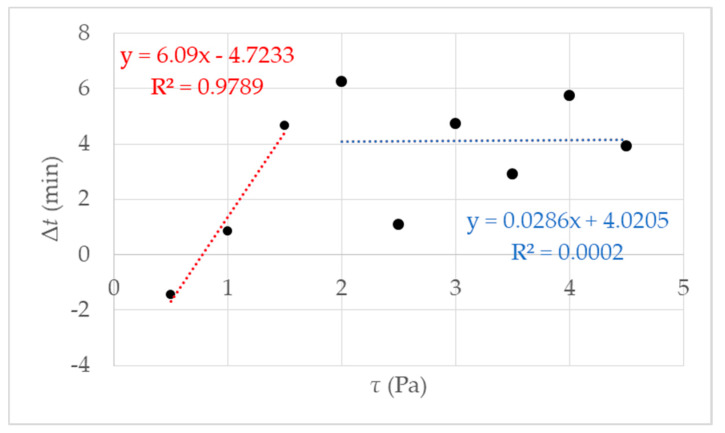
The figure shows the results of the rotational rheometry with different shear stress *τ* = 0.5 up to 4.5 Pa and an increasing gelation Δ*t*; the red fit is an example of increasing the gelling time until shear stress *τ* = 1.5 Pa, the blue fit can be regarded as a guide for the eye.

The proportion of liquid leads to the assumption that the reaction mixture is no longer completely polymerized, and the formation of a macroscopic network is prevented, i.e., gel formation is suppressed. Above the critical shear stress, a constant *tg* can be observed with an accuracy of ± 2 min. Accurately, gelation is suppressed. That is illustrated in Figure 5, and the exact value for the critical shear stress can be derived *τ_c_* = 1.5 Pa. With undisturbed gelling, we know that a macroscopic polymer network is formed after 20 min (*tgel*) [27]. We checked this value with simple tilting tests. With *tgel* = 20 min delta t is determined as the difference between *tg* and *tgel* (see Figure 1b). The results of the calculated delta t are presented in Figure 5 where delta *t* get a constant value of 4 ± 2 min from *τ_c_* = 1.5 Pa. The reason is the destruction or prevention of gelling due to the mechanical influence, a complete polymer network cannot form. This assumption is confirmed when the measurement geometry was opened after the measurement. The plate was covered with 40% liquid polymer solution at a shear stress of 1.5 Pa. With increasing shear stress, the liquid fraction also increased until it remained completely liquid. A linear increase of delta *t* until the critical *τ_c_* = 1.5 Pa (in Figure 5 the fit in red) and then reaching constant value above *τ_c_* with delta *t* 4 min ± 2 min (Figure 5 the blue fit). An exact determination of the gel point is no longer possible because there is no longer a macroscopic network. For the investigated NIPAm with Laponite^®^ XLS system, a critical value of *τ_c_* = 1.5 Pa was found. The following studies were performed at a temperature of 293 K and shear stress of *τ* ≤ 1.5 Pa, to guarantee the comparability with DLS under flow.

The findings of this study support the assumption that the gelation is a mechanism that can be strongly influenced by mechanical shear. Owing to this finding, further influencing factors were investigated. The repeatability was ±2 min by the determination of the gel point. With the following studies, we enhanced the measurement method for better comparability with the DLS. Due to the bits of knowledge that clay aggregates are formed at the beginning of polymerization, investigations of SP are from paramount importance.

**Figure 6:**
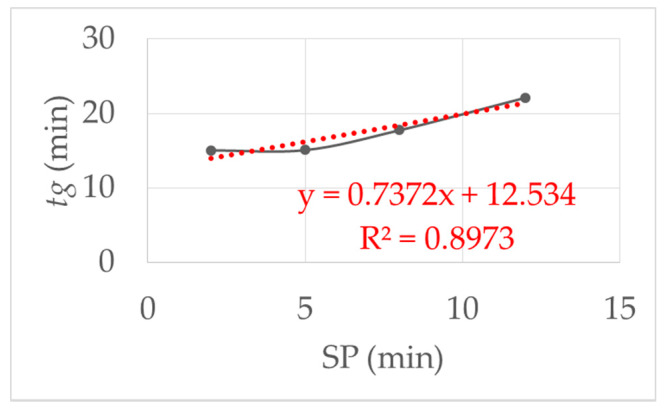
This figure shows the influence of the starting point delay (SP): linear increase in the gel time with increasing SP. SP was varied from 2, 5, 8 and 12 min with the same initial viscosity and shear stress *τ* = 1.5 Pa at 293 K.

The influence of the starting point delay (SP) was examined experimental. Figure 6 show the results when the SP was varied from 2, 5, 8 and 12 min with the same initial viscosity of the monomer solution. As the SP increased, the gelling time increased. The reason for this is the adsorption of the reactive components on the clay platelets, as described in [1]. By resting the reaction mixture, the prepolymerization phase was not disturbed, so that it took longer for the polymer chains to come together in a network. This led to an almost linear increase in the gelling time. 

Furthermore, the dependence of viscosity on gelation time is essential. Initial viscosity depends on the one hand, on temperature, and on the other hand, on the content of the solved reactants. Therefore we decided to variate the viscosity by variation of NIPAm quantity. We produced monomer solutions with double monomer content and investigated the influence of increasing viscosity on the gelation. The observed starting viscosity varies with different monomer solutions, even at the same concentration. The causes are the self-polymerization of NIPAm by atmospheric oxygen and temperature differences in the measuring geometry, although its temperature is controlled. The avoiding temperature influence could not be optimally guaranteed, even by preheating the sample in a water bath to measuring temperature. Due to the high consumption, the subsequent purchase of the starting materials is inevitable, and the open measuring geometry cannot prevent self-polymerization. Consequently, an SP of 2 min was always measured.

The doubling of the monomer content leads to an enormous increase in viscosity. An example of standard value with a viscosity of 2.6 mPa·s led to *tg* = 17.76 ± 1.4 min and double concentration with a viscosity of 25 mPa·s a *tg* = 24.43 ± 0.98 min was found. The gel time increased with increasing viscosity. The resulting higher viscosity slowed down the diffusion of the educts. Therefore, monomers could move through the mixture more slowly. Consequently, the polymerization was slower. We detected that during the aging of the NIPAm solution the viscosity increase, because of self-polymerization. At the beginning, day 1 the NIPAm–clay system with a viscosity of *η* = 2.7 mPa·s we found a *tg* = 15.08 ± 0.28 min and day 14 a NIPAm–clay system with *η* = 10 mPa·s and *tg* = 20.2 min ± 1.39 min was measured. Our data corroborated the self-polymerization of the monomer solution that led to chain growth and thus minimized crosslinking. This led to an extension of the network formation and the polymerization was slowed down. Therefore, the monomer solution was freshly prepared before each measurement to measure at an initial viscosity of 2.5–3.5 mPa·s. The rate-determining step in radical polymerization is the formation of radicals, the rate can be accelerated by increasing the initiator concentration. Persulfate gives two radicals, so doubling the initiator means four-times that much radicals. Accordingly, the gelation time became approximately 4 times shorter. See Table 1; these findings proved our hypothesis. An example for doubling the initiator concentration is shown in Table 1, which led to the faster radical formation and, thus, to a shortened gelation time. 

Our gelation time was within 10% of the theoretically expected value. The initiator concentration significantly affected the gelation time.

### 3.2. Dynamic Light Scattering

#### 3.2.1. Dynamic Light Scattering without Mechanical Load

**Figure 7:**
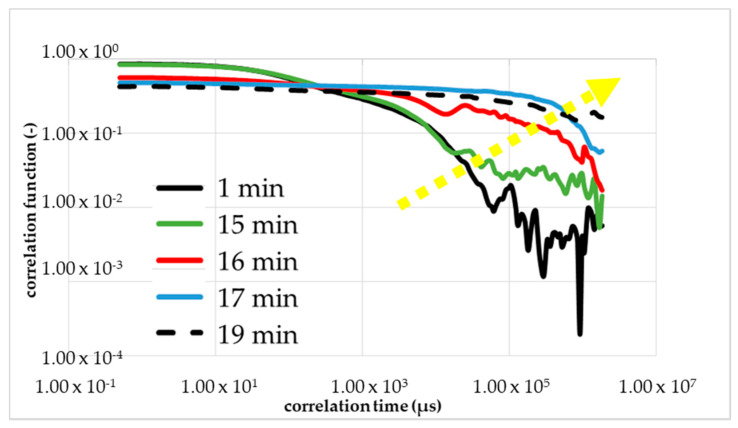
First calibration measurement with DLS to determine the gel point without shear. A change of TCF of NIPAm, PPS and TEMED solution during its gelation process occurred.

The comparison under increasing mechanical stress is made more difficult by the fluctuation in the viscosity of the monomer solution. Therefore, a single monomer solution was used with all further measurements, i.e., for the calibrations measurements with rheometry, DLS and the measurements with mechanical shear. Within Figure 7 was the first figure calibration measurement (first run) to determine the gel time with the DLS in the resting state (without a flow/shear). The shape of the correlation function changed between 16 and 17 min (red curve) after the start of the reaction (black curve). The blue curve corresponded to 1 min after the change in the shape and the green curve corresponded to 1 min before the change. The dashed line is the correlation function 20 min after the initiation. The yellow arrow shows the direction of the change in the curve shape, i.e., reaction order.

The results of the measurement were plotted double logarithmically. The calibrating measurement with DLS was performed at 293 K and a set starting point delay of 2 min. At the beginning, we observed the drop of the black line in Figure 7; that is what we expected for the colorless liquid monomer solution. After around 15 min, we see a linearization of the green line because of the local turbidity of the sample by the cluster forming.

**Figure 8:**
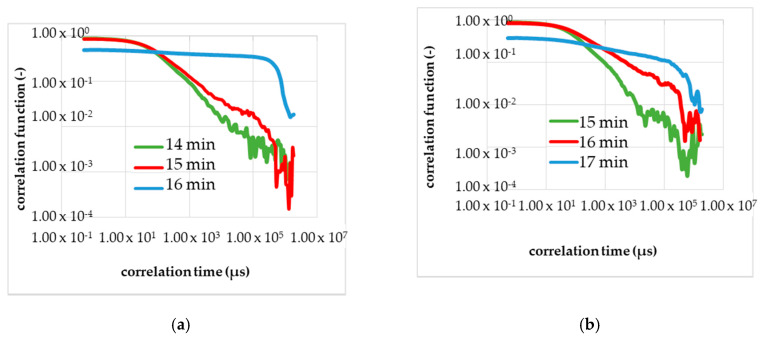
Calibration measurements (a—second run, b—third run) to determine the gel time with the DLS in the resting state (without a flow). (**a**) The shape of the correlation function changes in 16–17 min after the start of the reaction; red curve. The blue curve; corresponds to 1 min after the change in the shape, and the green curve corresponds to 1 min before the change. (**b**) The shape of the correlation function changes in 17–18 min after the start of the reaction; red curve.

Further, a sharp and short reduction of the optical transmittance during the polymerization to NC gels is known from Ferse et al. The shape of TCF red line with 17 min changes drastically from a falling exponential to linear behavior at the gelation threshold. At the gel point, the shape changed from a falling curve to a linear line. However, the gelling, the building of a macroscopic cluster, was not complete. Therefore, the blue line is shown, almost parallel to the dashed line, which is the measurement result of the final gel. We proved the reproducibility with a second and third calibration measurements presented in Figure 8a,b.

Figure 8a shows the results of the second calibration measurement in the resting state with the DLS, and Figure 8b, the third calibrating measurement. Analogously to Figure 7, the curve of the correlation function changed several minutes after the start of the chemical reaction with adding of the initiator. In Figure 8 is in each diagram, three correlation functions shown and plotted double logarithmically.

The green line is the TCF before the change in the shape appeared. The red line is the TCF at the gel point, measurable by the reduction of the optical transmittance. The blue line is given to demonstrate that the gelling process is not final and the red line marks the gel point. In Figure 8a the red line corresponds to the correlation function 16–17 min after the initiation, and Figure 8b shows the gel point in 17–18 min after the start.

The reproducibility of the measurement was observed within the conducted calibration. With the same monomer solution or the same viscosity, the identical gelation time was measured in three independent runs. The gelation time of 17 ± 1 min without mechanical influence serves as a reference for the measurement in the shear flow. Figure 7 and Figure 8 show that the shape of the curves (i.e., TCF) changes slowly and not suddenly, which corresponds to the process of the gelation, which is a phase transition.

#### 3.2.2. Dynamic Light Scattering under Mechanical Shear

By increasing the volumetric flow to 45 mL/min with the peristaltic pump, we were able to increase the mechanical shear in the measuring set up (Figure 3) of the DLS measurement set up under flow. First mixing of the reactants in a beaker. With the input of the reactants (through the valve), the measuring was started. The preparatory work: mixing reactants and inserting to the setup, was timed of 2 min, respectively starting the time delay.

**Figure 9:**
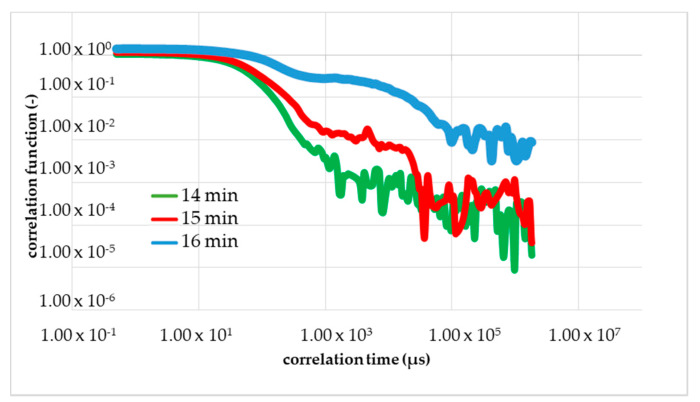
The result of DLS under flow at a volumetric flow of 45 mL/min. The shape of the correlation function changes in 15 min after the start of the reaction red curve. The blue curve corresponds to 1 min after the change in the shape and green curve corresponds to 1 min before the change.

Figure 9 shows the result of the DLS measurements under a shear flow at a volumetric flow of 45 mL/min. The volumetric flow in the hose led to a wall shear rate of approximately 120 1/s and was comparable to a shear stress of less than 0.5 Pa. An observed change in the correlation function corresponded to the time of approximately 15 min. The gel point at 15 min after initiation, earlier than expected. That is leading to the assumption that further influencing factors exist that influence or expedite the gelling.

### 3.3. Discussion

In this study, we first tried to specify the factors influencing the gel time. We showed the most important influencing factors such as the viscosity, which depends on the content and the temperature of the reactants. Besides, we improved or developed the ideal measurement conditions to obtain reproducible results. First we have to discuss the result of the DLS in the resting state. Unlike as expected, gelation appears in the resting state earlier, compared to *tgel* with 20 min [27]. I presumed that the laser light of DLS locally heated the sample. The radical polymerization was faster due to local heating of the sample, and local turbidity appears. We proved this with a laser *λ* = 632.8 nm He-Ne laser (Spectra Physics) with a maximum power output of 4.5 mW. By filling 1 mL reactants solution into the transparence cuvette and pointing the laser beam on the cuvette. In the area where the laser beam pointed to the solution, a local turbidity was visible. The turbidity disappeared 20 min after the initiation when the polymeric network was formed. This turbidity led to a change in the correlation function. We obtained by DLS under flow a gelation time of about 15 ± 1 min and by rheometry with a higher mechanical shear of about 15.3 min. The slight difference in the gelation time obtained by DLS to the rheometric characterizations could be accounted to the influencing factors mentioned in the shown studies. The initial viscosity was the influencing factor to the gelling time and respectively to the measured gel point. The viscosity depending on the temperature, e.g., differences in the sample temperature led to different initial viscosity. The sample must have the same temperature in each measurement. Therefore, a constant and controlled temperature is necessary during rheometric and DLS measurements with the Peltier unit of the rheometer guaranteed and the DLS has an integrated thermostat, too. However, the DLS measuring set up was self-made (see Figure 3) with no possibility to control the temperature of the sample in the hose. That could lead to fluctuations in temperature and following a different initial viscosity.

We illustrated the influence of fluctuation in viscosity with experiments with increased viscosity and resulting in extended gelling times. Not only temperature also the aging of the monomer solution led to an increase in viscosity. Based on this sensitivity analysis, the conditions of the measurements with the DLS are determined to receive comparable results. In [37] further explanation, answering the question of whether the “microscopic” gel point is the same as the “macroscopic” gel point determined using rheometry, is provided. The gel point of gelatine was evaluated in [37], where the sol–gel process took place due to the decrease of the temperature. In the context of the gelatin study, it was shown that the gelation temperature obtained by DLS and by rheometry corresponded to each other. The results corresponded with the accuracy of 1 K. In our current work, we could conclude that the effect of mechanical shear on gelation time, determined by DLS and rheometry, was consistent. Nonetheless, DLS and rheometry were comparable. Due to the process-related differences, there may be deviations in the measurement result. With the DLS, only partial temperature-controlled measurements were possible under flow, because the hose with the sample was outside of the DLS (see Figure 3) hose. Moreover, the non-uniform cross-section in the cuvette led to a shear gradient and thus to different shear rates. Compared to the rheometer, a significantly higher shear rate could be assumed. Since *tg* = 15 min with DLS corresponded to a shear rate of 1.5 Pa.

## 4. Conclusions

In the present work, the viscosity increase using rotational rheometry, as well as the correlation function using the DLS during the radical gelation, were determined on the system N-isopropylacrylamide (NIPAM)/clay in a concentration ratio of 10.4/7 wt %. The initiator concentration was chosen in such a way that rapid gelation was to be expected, and thus measuring times were reduced from 120 min in previous work [27] to 20 min in this work to observe the sol–gel conversion. Before measurements could be carried out at the DLS, previous rheometric measurements carried out to identify and eliminate the influencing factors on the gelation. This is temperature, viscosity, aging and quantity of the educts. The determination of the gelation point with the DLS was carried out in the resting state and under shear flow with a wall shear rate corresponding to the shear stress used in the rheometrical measurements. Results obtained with these different methods were comparable, if the same monomer solution, on the same day, at the same temperature was used. However, there is still a need to better understand the influence the mechanical stress during gelling on the resulting structure–property relationships. An approach is to study and compare the mechanical properties of finished gels that are treated with and without mechanical stress. It should not be forgotten that the clay content influences the network of the resulting gel. Therefore, investigations with different clay contents are necessary. The reproducibility of the exact gelation time is negatively influenced by atmospheric oxygen. Therefore, all measurements should be repeated in the absence of oxygen, with a technically optimized set up. This can reduce the self-polymerization of the NIPAm. Furthermore, temperature influence must be examined in detail. 

## Figures and Tables

**Table 1:** Influence of the initiator concentration on the gelation time.

	At Initial Initiator Concentration	At Double Initiator Concentration
*tg* $\bar{x}$	17.8 ± 1.57 min	4.86 ± 0.35 min

^1^*T* = 293 K, *τ* = 1.5 Pa.
